# Supplementary material for: Dynamics of fMRI patterns reflect sub-second activation sequences and reveal replay in human visual cortex
Source: Nat Commun. 2021 Mar 19;12:1795. doi: 10.1038/s41467-021-21970-2 (PMC7979874; doi:10.1038/s41467-021-21970-2)
Supplement: Supplementary file 1 — Supplementary information [file 41467_2021_21970_MOESM1_ESM.pdf]

Dynamics of fMRI patterns reflect sub-second activation sequences and reveal replay in human visual cortex - Supplementary Information -

Lennart Wittkuhn[1,2*] and Nicolas W. Schuck[1,2*]

[1]Max Planck Research Group NeuroCode, Max Planck Institute for Human Development, Berlin, Germany

[2]Max Planck UCL Centre for Computational Psychiatry and Ageing Research, Berlin, Germany

[*]Corresponding authors, email wittkuhn@mpib-berlin.mpg.de and schuck@mpib-berlin.mpg.de

Supplementary Notes

Additional behavioral results Attentive processing of the visual stimuli was a prerequisite to study the evoked activation patterns in visual and ventral temporal cortex. We therefore excluded all participants that performed below chance on either or both the repetition and sequence trials of the task. To this end, we removed all participants with a mean behavioral accuracy below the 50% chance level from all further analyses (Supplementary Fig. S1a). We also compared the relative proportion of misses and false alarms for each of the eight fMRI task runs in the experiment. To this end, we conducted a LME model with trial type (miss, false alarm), session (first, second) and session run (run 1–4) as fixed effects and included by-participant random intercepts and slopes. As shown in Supplementary Fig. S1b, misses ($M = 0.55\%$) consistently occurred more frequently than false alarms ($M = 0.30\%$), $F_{1,501.00} = 4.12$, $p = .043$, which was consistent across task runs (no effects of session or run, $ps \leq .703$). Our classification was performed using a leave-one-run-out approach. In order to examine whether the accuracy of behavioral performance on slow trials was stable across all task runs of the study, we conducted a LME model that included the eight task runs as the fixed effect of interest as well as random intercepts and slopes for each participant. The results showed no effect of task run indicating that the accuracy of behavioral performance was relatively stable across task runs, $F_{1,92.72} = 0.13$, $p = .72$ (Supplementary Fig. S1c). We examined whether mean behavioral accuracy on sequence trials was influenced by either the sequence speed or the serial position of the cued target image. A LME model including the sequence speed as a fixed effect and by-participant random intercepts and slopes indicated slightly lower but clear above-chance performance if the sequences were displayed at faster speeds, $F_{1,35} = 4.27$, $p = .046$ (Fig. 1f). A separate LME model including the target position as a fixed effect and by-participant random intercepts and slopes indicated lower but above-chance performance if the target image appeared at earlier serial positions, $F_{1,42.02} = 9.92$, $p = .003$ (Supplementary Fig. S1d). We focused the analysis of repetition trials on the forward and backward interference condition in the main text, but also examined performance for all intermediate repetition conditions and conducted a LME model with repetition condition as a fixed effect and by-participant random intercepts and slopes. Mean behavioral performance decreased with the number of second item repetitions, $F_{1,39} = 57.43, p < .001$ (Supplementary Fig. S1e). A series of eight one-sided one-sample t-tests indicated that for all repetition conditions mean behavioral accuracy was above the 50% chance level ($t_{35} \geq 2.35$, $ps \leq .012$, FDR-corrected; $ds \geq 0.39$).

Decoding in the hippocampus is at chance level We also conducted a separate leave-one-run-out classification analysis to decode the five stimulus categories from activation patterns in the hippocampus. To this end, the same decoding approach was used but activity patterns were extracted from an anatomical ROI centered on the hippocampus. The ROI was based on the same automated anatomical labeling of brain surface reconstructions from the individual T1w reference images that were used to create the anatomical masks of occipito-temporal brain regions. No GLM-based feature selection was performed on activity patterns from the hippocampus. Using the hippocampal masks in the leave-one-run-out cross-validation approach revealed that the average classification accuracy ($M = 20.52\%$, $SD = 1.49\%$, range $= 17$–24%) did not differ from the chance baseline of 20%, $t_{(35)} = 2.10$, 95% CI [20.02, 21.03], $p = .05$, $d = 0.35$ (two-sided one-sample t-test, no multiple comparisons; see Supplementary Fig. S2). The implications of this finding are further discussed in the main manuscript.

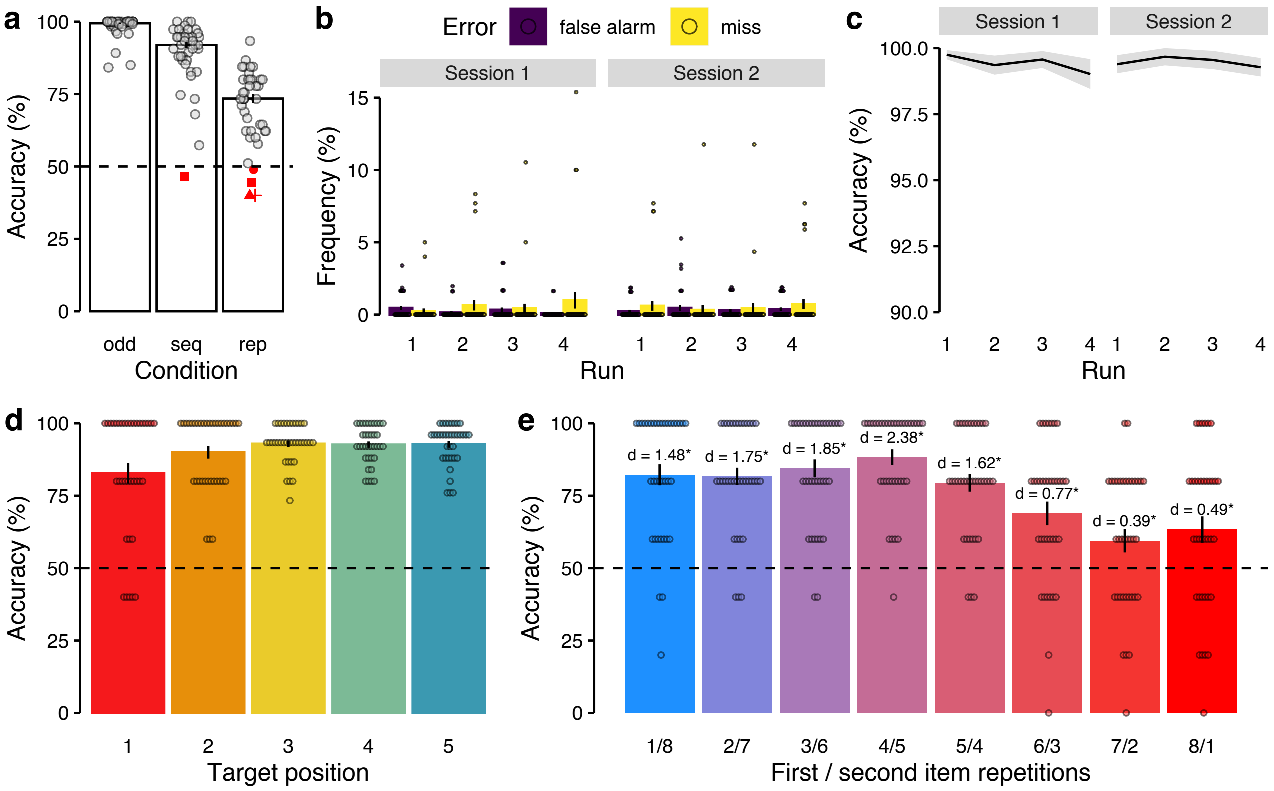

Supplementary Figure S1: Additional behavioral results. **(a)** Mean behavioral performance (in %; y-axis) for the three trial conditions (x-axis). Dots / symbols represent mean data of a single participant with below-chance performance colored in red. Note, that the errorbars were derived from data of $N = 36$ human participants after participants with below-chance performance were excluded and indicate mean values ±1 SEM ($N = 36$, $ts \geq 14.50$, $ps \leq .001$, d's ≥ 2.42, one-sided one-sample t-test per condition, no correction for multiple comparisons) **(b)** Mean frequency of incorrect slow trials (in %; y-axis) across the four task runs (x-axis) of each study session (panels), separately for false alarms (violet bars) and misses (yellow bars; $N = 36$, $F_{1,501.00} = 4.12$, $p = .043$ for main effect of error type, LME model). **(c)** Mean behavioral accuracy on slow trials (in %; y-axis) across the four task runs (x-axis) of each study session (panels; $N = 36$, $F_{1,92.71} = 0.13$, $p = .72$ for main effect of task run, LME model). **(d)** Mean behavioral accuracy on sequence trials (in %; y-axis) as a function of serial position of the target stimulus (x-axis; $N = 36$, $F_{1,42.02} = 9.92$, $p = .003$ for main effect of target position, LME model). **(e)** Mean behavioral accuracy on repetition trials (in %; y-axis) for all repetition conditions (x-axis) compared to the 50% chance-level ($N = 36$, $ts \geq 2.35$, $ps \leq .012$, d's ≥ 0.39, eight one-sided one-sample t-tests, FDR-corrected). Asterisks indicate $p < .05$, FDR-corrected. Effect sizes are indicated by Cohen's d. Horizontal dashed lines (in a, d, e) indicate 50% chance level. Errorbars (in a, b, d, e) and shaded areas (in c) represent ±1 SEM. All statistics have been derived from data of $N = 36$ human participants. Source data are provided as a Source Data file.

Spatial correlations between classifier patterns According to previous fMRI studies that investigated the neural representations of visual objects, corresponding multi-voxel patterns are often found to be widely distributed and largely overlapping within occipito-temporal brain regions [e.g., 55, 58–60]. To investigate the spatial distribution and overlap between the voxel activation patterns of the five visual stimuli used in our study, we visually examined their mean average activation patterns (an example from one participant is shown in Supplementary Fig. S3). Overall, the five stimuli appeared to activate a mix of overlapping and non-overlapping sets of voxels. In order to quantify this impression, we calculated the mean spatial correlation between voxel-activations for each participant. These analyses indicated that classifier patterns were slightly negatively correlated, with Pearson's correlations ranging from $r = .02$ for the correlation between cat and face to $r = -.44$ for the

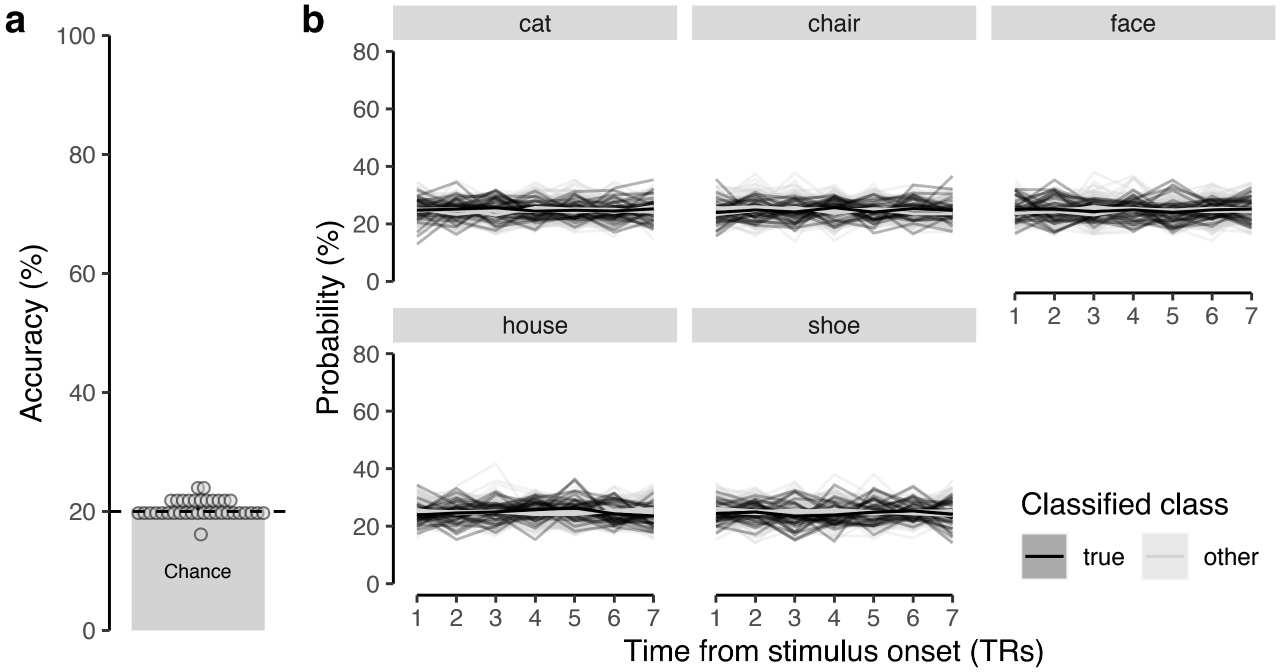

Supplementary Figure S2: Classification accuracy in the hippocampal mask. **(a)** Cross-validated classification accuracy in decoding the five unique visual objects in hippocampal data during task performance (in %; $N = 36$, $t_{(35)} = 2.10$, 95% CI [20.02, 21.03], $p = 0.05$, $d = 0.35$, one two-sided one-sample t-test, no multiple comparisons). Chance level is 20% (dashed line). Each dot corresponds to averaged data from one participant. The errorbar represents ±1 SEM. **(b)** Time courses (in TRs from stimulus onset; x-axis) of probabilistic classification evidence (in %; y-axis) for all five stimulus classes. No probability increases for any stimulus presented (black lines) on a given trial (gray panels) were found. Each line represents one participant. All statistics have been derived from data of $N = 36$ human participants. Source data are provided as a Source Data file.

correlation between cat and house (see Supplementary Table S1 below).

Supplementary Table S1: Average correlation between average spatial patterns associated with each image category. Source data are provided as a Source Data file.

	Cat	Chair	Face	House	Shoe
Shoe	-.33	-.16	.29	-.15	1
House	-.44	-.20	-.37	1	
Face	.02	-.31	1		
Chair	-.23	1			
Cat	1				

Additional information on single event and event sequence modeling As reported in the main text, we described multivariate decoding time courses on slow trials by a sine wave response function that was fitted to the decoding time courses of all participants separately. Evaluating a single sine wave response function for three randomly selected example participants based on the individually fitted parameters indicated that the response functions capture the individual participant data well (Supplementary Fig. S4a). Based on the mean parameters across all participants we derived the mean response functions for each stimulus class which looked qualitatively similar (Supplementary Fig. S4b).

Additional results for sequence trials As reported in the main text, we investigated whether sequence order was evident in the relative pattern activation strength within a single measurement (i.e., within a single TR) and quantified sequential ordering by the slope of a linear regression between serial events and their classification probabilities. In addition, we repeated the same analysis using two different indices of linear association which produced qualitatively similar results. First, using ranked correlation coefficients (Kendall's τ) between the serial event position and their classification probabilities as the index of linear association, we also found significant forward ordering in the forward period at sequence speeds of 128, 512 and 2048 ms (ts ≥ 2.13; ps $\leq .05$, FDR-corrected; ds ≥ 0.36) and significant backward ordering in the backward period for all speed conditions (ts ≥ 4.24; ps $< .001$, FDR-corrected; ds ≥ 0.71; Supplementary Fig. S5a–b). Second, we ordered the probabilities at every TR and calculated the mean step size (i.e., difference) between the probability-ordered event positions. Again, this analysis revealed qualitatively similar results, as we found significant forward ordering in the forward period at sequence speeds of 128, 512 and 2048 ms (ts ≥ 2.25; ps $\leq .04$, FDR-corrected; ds ≥ 0.37) and significant backward ordering in the backward period for all speed conditions (ts ≥ 4.73; ps $< .001$, FDR-corrected; ds ≥ 0.79; Supplementary Fig. S5c–d).

Next, we analyzed the time courses of linear associations in more detail. Specifically, for each index of linear association, we tested for sequentiality at every time point (i.e., at every TR) and conducted a series of two-sided one-sample t-tests comparing the sample mean at every time point against zero (the expectation of no order information). All p values were adjusted for multiple comparisons by controlling the FDR across all time-points within the forward and backward period and speed conditions (38 comparisons in total). This analysis produced consistent results for each index of linear association that was tested. For the mean regression slopes, this analysis revealed significant forward sequentiality at earlier time points for all speed conditions (TR 3 at 128 ms, $t_{35} = 2.37$, $p = .04$, $d = 0.40$; TRs 3–4 at 512 ms, ts $= 6.16$, ps $< .001$, ds ≥ 1.03; TRs 3–7 at 2048 ms, ts $= 7.78$, ps $< .001$, ds ≥ 1.03; all ps FDR-corrected for 38 comparisons) except the 32 and 64 ms speed condition (ps $\geq .08$). Furthermore, we found significant backward sequentiality at later time points for all speed conditions (TRs 5–7 at 32 ms, ts $= 2.77$, ps $\leq .02$, ds ≥ 0.46; TRs 5–6 at 64 ms, ps $\leq .02$, ds ≥ 0.46; TRs 6–7 at 128 ms, ts $= 3.53$, ps $\leq .003$, ds ≥ 0.59; TRs 6–7 at 512 ms, ts $= 6.41$, ps $< .001$, ds ≥ 1.07; TRs 8–12 at 2048 ms, ts $= 4.21$, ps $< .001$, ds ≥ 0.70; all ps FDR-corrected for 38 comparisons; S6a). As can be seen in Supplementary Figs. S6b–d these results were qualitatively similar for all other indices of linear association tested (rank correlation coefficients and mean step size between probability-ordered event positions).

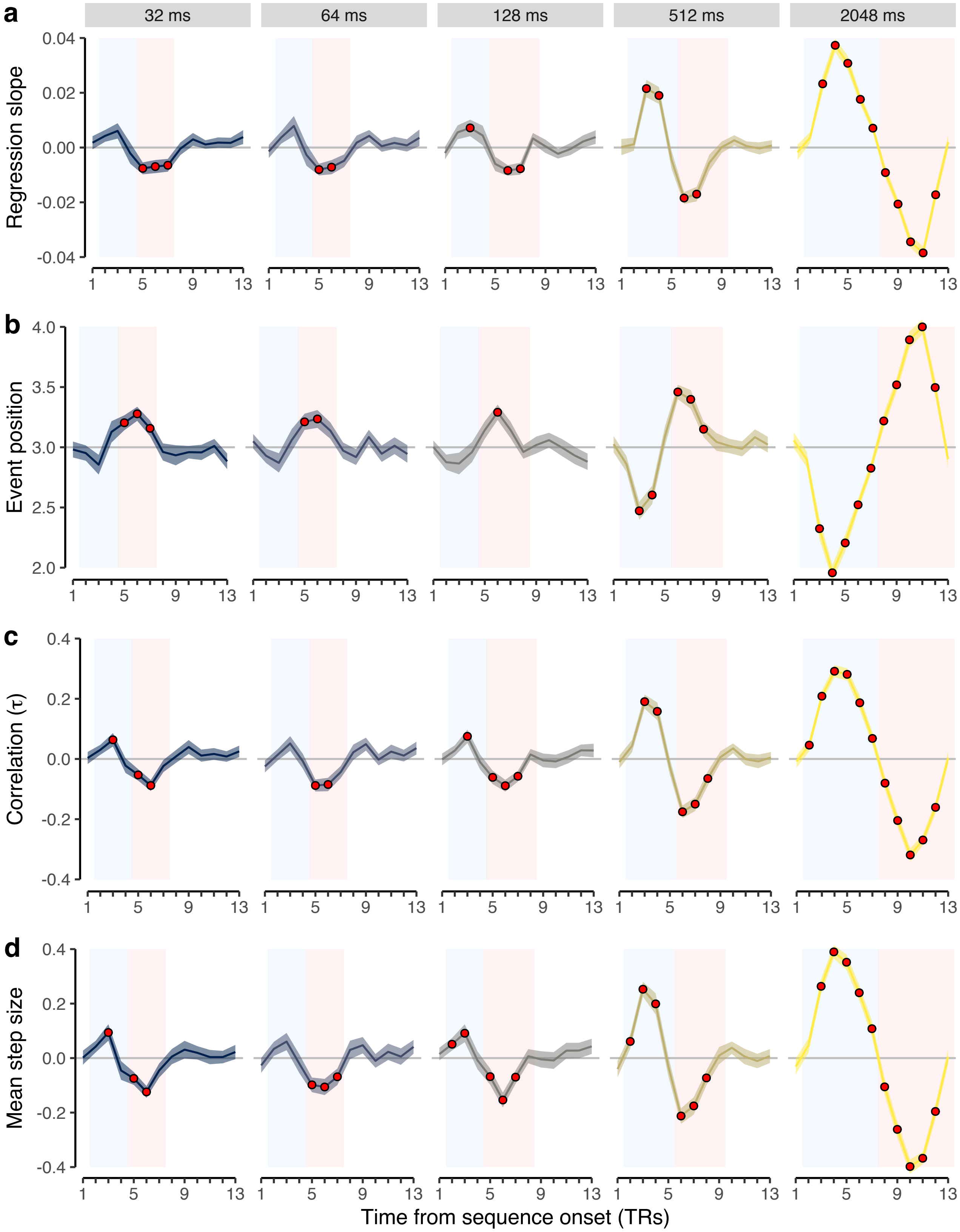

Supplementary Figure S6: Classification time courses on sequence trials. Time courses (in TRs from sequence onset; x-axis) of **(a)** mean linear regression coefficients (slope), **(b)** mean decoded serial event position with maximum probability for each sequence presentation speed (in ms; panels / colors), **(c)** mean correlation coefficients (Kendall's τ), and **(d)** mean step size between probability-ordered within-TR events. Shaded areas represent ± 1 SEM. All statistics have been derived from data of $n = 36$ human participants. The blue and red rectangles indicate forward and backward period, respectively. Red dots indicate significant differences from baseline (horizontal gray line at zero; all $ps \leq .05$, FDR-corrected for 38 comparisons; two-sided one-sample t-tests). 1 TR = 1.25 s. Source data are provided as a Source Data file.

As reported in the main text, we verified that the sequentiality effects observed on sequence trials (Fig. 3b) are not only driven by the event with the maximum probability but that sequentiality is also present if the event with the maximum probability is removed. Examining the mean slope coefficients within the expected forward and backward period (adjusted by considering only four sequence events) after removing the event with the maximum probability showed that we could still find evidence for sequential ordering (Supplementary Fig. S7a). Significant forward ordering in the forward period was still evident at sequence speeds of 512 and 2048 ms ($ts \geq 3.31$; $ps \leq .004$, ten two-sided one-sample t-tests, FDR-corrected; $ds \geq 0.55$) and significant backward ordering in the backward period for all speed conditions ($ts \geq 3.74$; $ps \leq .002$, ten two-sided one-sample t-tests, FDR-corrected; $ds \geq 0.62$; Supplementary Fig. S7b) except the 32 and 128 ms speed conditions ($p \geq .20$). The main analysis reported in the Results section highlighted an apparent asymmetry in detecting forward and backward sequentiality. To determine the extent to which this asymmetry was driven by the first or last item in the sequence, we conducted two additional control analyses by either removing the first or last sequence item from the analysis. Removing the first sequence item did not change the observed sequentiality effects qualitatively (Supplementary Fig. S7c) as we still found significant forward ordering in the forward period at sequence speeds of 512 and 2048 ms ($ts \geq 5.72$; $ps < .001$, FDR-corrected; $ds \geq 0.95$) and significant backward ordering in the backward period for all speed conditions ($ts \geq 2.65$; $ps \leq .02$, ten two-sided one-sample t-tests, FDR-corrected; $ds \geq 0.44$; Supplementary Fig. S7d). Removing the last sequence item, in contrast, made any significant sequentiality disappear for speed conditions of 128 ms or faster ($p \geq .27$), while forward and backward sequentiality were still evident at sequence speeds of 512 ms and 2048 ms ($ts \geq 3.55$; $ps \leq .05$, ten two-sided one-sample t-tests, FDR-corrected; $ds \geq 0.59$; Supplementary Figs. S7e–f).

Additional analyses of repetition trials We conducted two additional analyses for the data on repetition trials. First, we analyzed the effect of event duration (number of repetitions) on event probability in more detail by calculating the average event probability for each event type (first, second, and averaged non-sequence) as a function of event duration (number of repetitions). Importantly, while we focused only on the two repetition conditions with the highest degree of interference before, we now also included the data from all intermediate repetition trial types. As before, we averaged the probabilities for each serial event type but this time as a function of how often each item type was repeated in any given trial. Then, in order to test how likely we were in decoding each serial event type (first, second, non-sequence), when each item was only shown briefly once, we conducted three independent pairwise two-sample t-tests comparing the mean probabilities of all three event types with one another (correcting for multiple comparisons using Bonferroni correction). The results reported in the main text focused on the two repetition conditions with the strongest expected effects of forward and backward interference. Additionally, we characterized the effect of event duration (number of repetitions) in more detail by analyzing the average probability of event types (first, second, non-sequence) as a function of event duration also for all intermediate repetition conditions. The results revealed a main effect of event type (first, second, non-sequence), $F_{2,278.97} = 23.99$, $p < .001$ and event duration (number of repetitions), $F_{1,58.73} = 183.10$, $p < .001$ as well as an interaction between event type and event duration, $F_{2,753.00} = 52.53$, $p < .001$ (see Supplementary Fig. S8). In order to further characterize the origin of this interaction, we also conceived a reduced model that did not include the data from non-sequence events. The results of this reduced model again showed a main effect

of event type (first, second), $F_{1,350.19} = 12.11$, $p < .001$ and event duration (number of repetitions), $F_{1,125.87} = 187.86$, $p < .001$ but no interaction between event type and event duration, $F_{1,501.90} = 0.10$, $p = .75$. If only shown briefly, the second event had a mean probability ($M = 17.07\%$, $SD = 5.42\%$) that was higher than for the first event ($M = 13.50\%$, $SD = 6.04\%$), $t_{(35)} = 2.45$, $p = .02$ and the averaged non-sequence items ($M = 7.75\%$, $SD = 2.93\%$), $t_{(35)} = 8.98$, $p < .001$ while the average probability of the first event was also higher compared to the out-of-sequence items, $t_{(35)} = 5.53$, $p < .001$ (all ps were adjusted for six multiple comparisons, using the Bonferroni correction). If the event duration was prolonged (eight consecutive repetitions) the second event had a mean probability ($M = 31.63\%$, $SD = 6.94\%$) that was significantly different from the first event ($M = 26.87\%$, $SD = 8.34\%$), $t_{(39)} = 2.59$, $p = .01$ and the averaged non-sequence items ($M = 7.69\%$, $SD = 2.69\%$), $t_{(35)} = 18.96$, $p < .001$ while the average probability of the first event was also higher compared to the non-sequence items, $t_{(35)} = 12.52$, $p < .001$ (all ps were adjusted for six multiple comparisons, using the Bonferroni correction). These effects were attenuated but qualitatively similar when data from all TRs were considered.

We asked whether we would be more likely to decode items that were part of the sequence actually shown to participants (within-sequence items) as compared to items not part of the sequence (out-of-sequence items). To this end, we assessed if the serial events 1 and 2 were more likely to be decoded in the repetition trials than other events. As before, we identified the item with the highest classifier probability at every TR of each trial and then calculated the relative frequency of each item in the decoded sequence of events. These frequencies were then averaged separately for each repetition condition across all trials and participants. Next, using paired t-tests, we performed two statistical tests: First, we tested how well we were able to decode a single briefly presented item in a 32 ms sequence compared to items that were not presented, when the item is followed by a statistical representation that could mask its activation pattern (short → long trials). Second, we tested how well we were able to decode a single briefly presented item (first serial event) in a 32 ms sequence compared to items that were not part of the sequence, when the item (last serial event) is followed by a random statistical signal, for example, during an ITI (long → short trials).

Analyzing the average proportion of decoded serial events across all TRs for the *backward interference* and *forward interference* conditions separately revealed a main effect of serial event type (first, second, averaged out-of-sequence), $F_{2,234} = 40.70, p = 6.80 \times 10^{-16}$. No main effect of repetition condition (short → long versus long → short) was found, $F_{1,234} = 0.08, p = .78$, but an interaction between serial event position and repetition condition, $F_{2,234} = 23.92, p = 3.54 \times 10^{-10}$ (see Fig. 4e). Post-hoc comparisons indicated that in the short → long condition the longer second event had a higher frequency ($M = 29.0\%$) compared to the out-of-sequence ($M = 17.4\%$) as well as the short, first event ($M = 18.9\%$, $ps < .0001$). The short first event did not differ from the out-of-sequence events ($p = .47$, Tukey-correction for three comparisons). In the long → short condition, in contrast, there was no difference between the long first ($M = 24.6\%$) and short second event ($M = 22.3\%$, $p = .17$, Tukey-correction for three comparisons) but significant differences between both within-sequence items and the averaged out-of-sequence ($M = 17.7\%$) items (both $ps < .001$, Tukey-correction for three comparisons).

Analyzing the mean probability for the three event types (first, second, and out-of-sequence events) on repetition trials as a function of the absolute event occurrence per trial using data from all 13 TRs revealed a main effect of event type (first, second, out-of-sequence), $F_{2,915} = 14.31, p < .001$ and event

duration (number of repetitions), $F_{1,915} = 68.97, p < .001$ as well as an interaction between event type and event duration, $F_{2,915} = 17.90, p < .001$ (see Fig. 4d). In order to further characterize the origin of this interaction, we also conceived a reduced model that did not include the data from out-of-sequence events. The results of this reduced model again showed a main effect of event type (first, second), $F_{1,597} = 10.92, p = .001$ and event duration (number of repetitions), $F_{1,597} = 78.92, p < .001$ but no interaction between event type and event duration, $F_{1,597} = 0.18, p = 0.68$. If only shown briefly, the second event had a mean probability ($M = 14.41\%$, $SD = 4.53\%$) that was higher than for the first event ($M = 12.02\%$, $SD = 4.78\%$), $t_{(39)} = 2.46, p = .03$ and the averaged out-of-sequence items ($M = 10.28\%$, $SD = 2.88\%$), $t_{(39)} = 5.80, p < .001$ while the average probability of the first event was also higher compared to the out-of-sequence items, $t_{(39)} = 2.52$, $p = .03$ (all p values were adjusted for six multiple comparisons, using the FDR correction). If the event duration was prolonged (eight consecutive repetitions) the second event had a mean probability ($M = 19.37\%$, $SD = 6.44\%$) that was not significantly different from the first event ($M = 16.54\%$, $SD = 4.75\%$), $t_{(39)} = 2.27, p = .06$ but from the averaged out-of-sequence items ($M = 9.75\%$, $SD = 3.05\%$), $t_{(39)} = 9.36, p < .001$ while the average probability of the first event was also higher compared to the out-of-sequence items, $t_{(39)} = 7.99, p < .001$ (all p values were adjusted for six multiple comparisons, using the FDR correction).

We also analyzed the trial-wise proportion of transition types between consecutively decoded events using data from all 13 TRs following stimulus onset. This analysis revealed that in the short $\rightarrow$ long condition the mean trial-wise proportion of forward transitions ($M = 6.50$) was higher than the mean proportion of outward transitions ($M = 2.48$), $t_{(39)} = 4.82, p < .001$ and also differed from the mean trial-wise proportion of outside transitions ($M = 1.28$), $t_{(39)} = 6.14, p < .001$ (all p values were corrected for four comparisons using Bonferroni correction; see Fig. 4f). Similarly, in the long $\rightarrow$ short condition, the mean trial-wise proportion of forward transitions ($M = 6.80$) was higher than the mean proportion of outward transitions ($M = 2.58$), $t_{(39)} = 6.11, p < .001$ and also differ compared to the mean trial-wise proportion of outside transitions ($M = 1.18$), $t_{(39)} = 7.71, p < .001$ (all p values were corrected for four comparisons using Bonferroni correction).

Repeating analyses of repetition trials using data from all TRs As reported in the main text, we focused the analyses of repetition trials on data from a relevant period of six TRs (from the second to the seventh TR) and the two trial conditions with maximum forward and backward interference, respectively. Here, we report results of the same analyses repeated using data from all TRs. The estimated probabilities of each stimulus class given the data for all repetition conditions are shown in Supplementary Fig. S9. Analyzing the mean probabilities of the different event types (first, second, out-of-sequence) using data from all TRs (see Supplementary Fig. S10a) revealed qualitatively similar results. Event type still influenced the average decoding probability, $F_{2,54.79} = 41.67$, $p < .001$ (see Supplementary Fig. S10b). Post-hoc comparisons indicated that sequence items had a higher mean probability than out-of-sequence (9.94%) items (both ps $< .001$, Tukey-correction for three comparisons), while the second (16.73%) and first (14.42%) within-sequence event type also differed ($p = .045$, Tukey-correction for three comparisons). Repeating the analysis for the forward and backward interference conditions using data from all TRs again revealed smaller but qualitatively similar effects, with a main effect of event type (first, second, out-of-sequence), $F_{2,44.29} = 55.22$, $p < .001$, an interaction between event type and duration, $F_{2,140.000} = 40.38$, $p < .001$, and no main

effect of duration (number of repetitions), $F_{1,116.04} = 0.15$, $p = .70$ (see Supplementary Fig. S10c). Post-hoc comparisons indicated that in the forward interference condition the longer second event had a higher probability (19.32%) compared to both the out-of-sequence ($M = 10.11\%$) and the short, first event ($M = 11.94\%$, ps $< .001$, Tukey-correction for three comparisons). As reported in the main text, when using data from all TRs, the short first event did not differ from the out-of-sequence events ($p = .09$, Tukey-correction for three comparisons). In the backward interference condition, in contrast, there was a significant difference between the long first (16.91%) and short second event (14.15%, $p = .04$, Tukey-correction for three comparisons) as well as significant differences between both within-sequence items and the averaged out-of-sequence (9.77%) items (ps $< .001$, Tukey-correction for three comparisons). We also repeated the analysis investigating trial-wise proportions of transitions between consecutively decoded events using data from all TRs. Based on the full transition matrix (see Supplementary Fig. S10e), this analysis revealed qualitatively similar effects (Supplementary Fig. S10d): Forward transitions (3.84%) between the two sequence items were as frequent as outward transitions (2.74%, $t_{(35)} = 2.61$, $p = .05$, Bonferroni-corrected for four comparisons) but more frequent than outside transitions (2.32%, $t_{(35)} = 2.61$, $p = .02$, Bonferroni-corrected for four comparisons) in the forward interference condition. The same was true for the backward interference condition (forward transitions: 4.54%; outwards transitions: 2.97%; outside transitions: 2.34%, all ts ≥ 3.56, all ps $< .001$; Bonferroni-corrected for four comparisons).

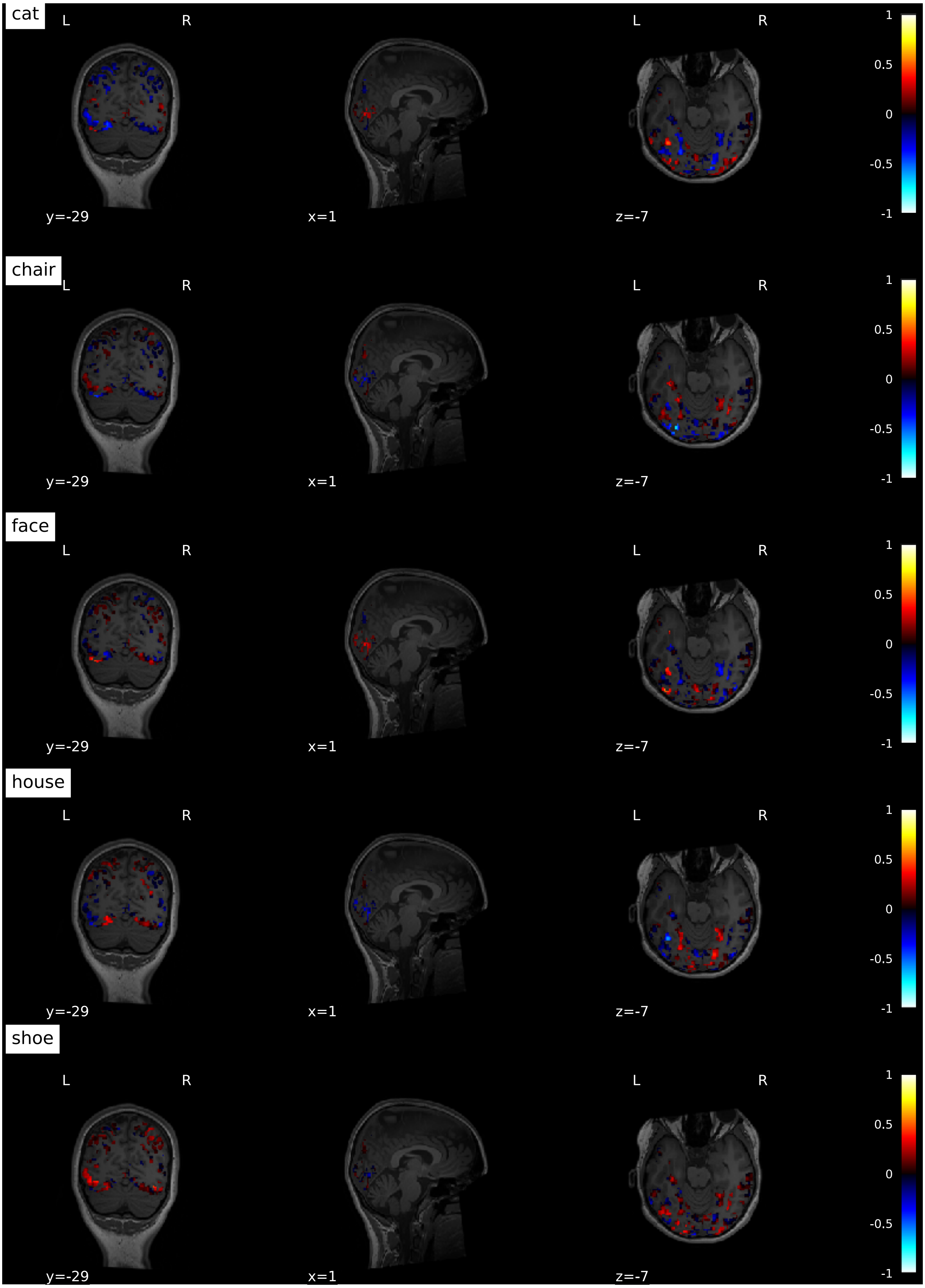

Supplementary Figure S3: Spatial distribution of mean voxel activations in one example participant for five stimuli Averaged patterns of voxel activations used for multivariate pattern analysis (colors indicate z-scored voxel activations) for the five decoded stimuli (horizontal panels) in one example participant (`sub-01`) shown against the participant's individual defaced structural scan.

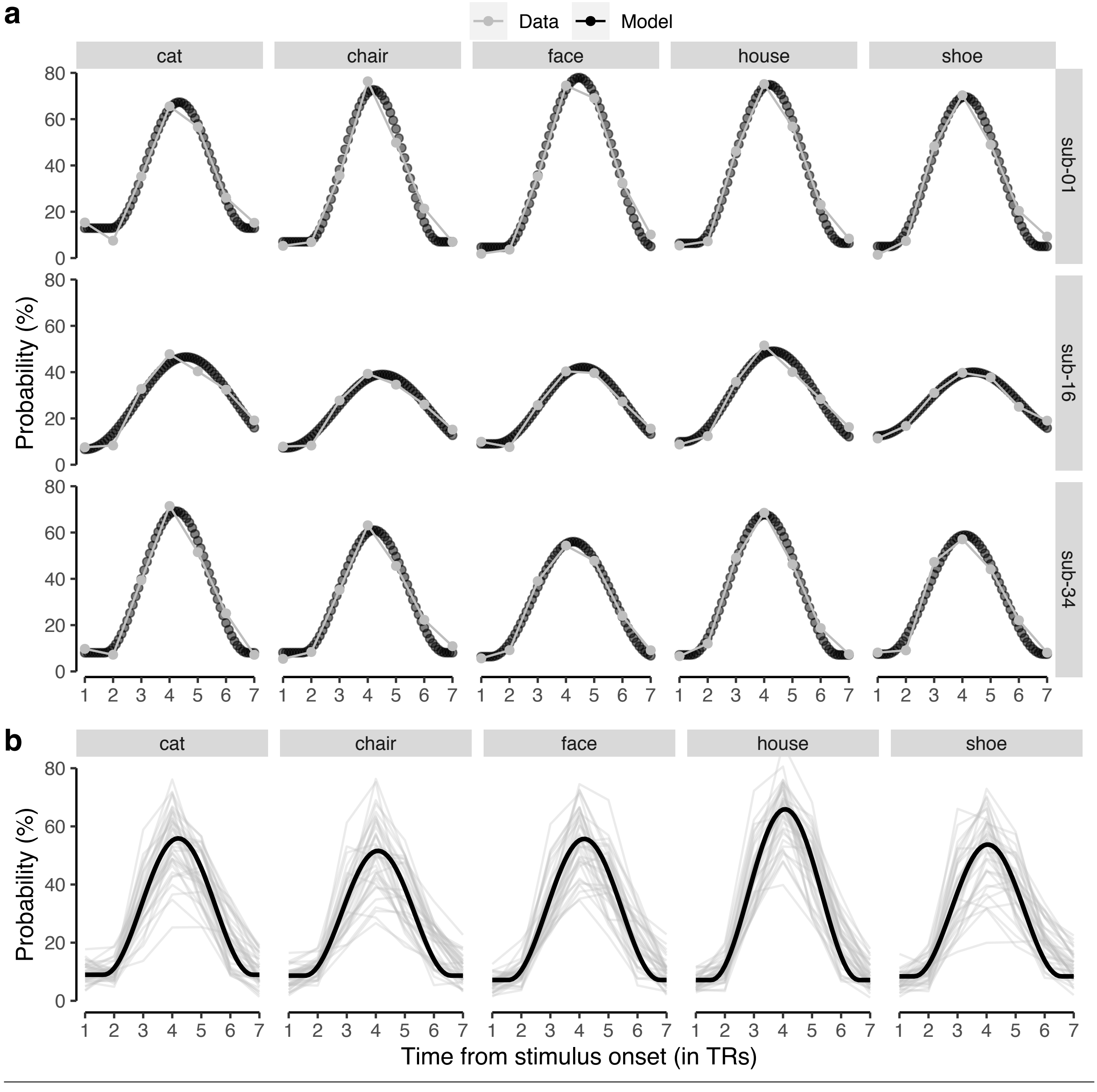

Supplementary Figure S4: Individual fits of sine wave response function to probabilistic classifier evidence. **(a)** Time courses (in TRs from stimulus onset; x-axis) of probabilistic classifier evidence (in %; y-axis) generated by the sine wave response function with fitted parameters (black dotted line) or the true data (gray line and dots) separately for the five stimulus classes (vertical panels) and three randomly chosen example participants (horizontal panels). **(b)** Time courses (in TRs from stimulus onset; x-axis) of mean probabilistic classifier evidence (in %; y-axis) averaged separately for each participant (gray semi-transparent lines) and stimulus class (vertical panels) or predicted by the sine wave response model based on fitted parameters averaged across all participants (black line). 1 TR = 1.25 s. Source data are provided as a Source Data file.

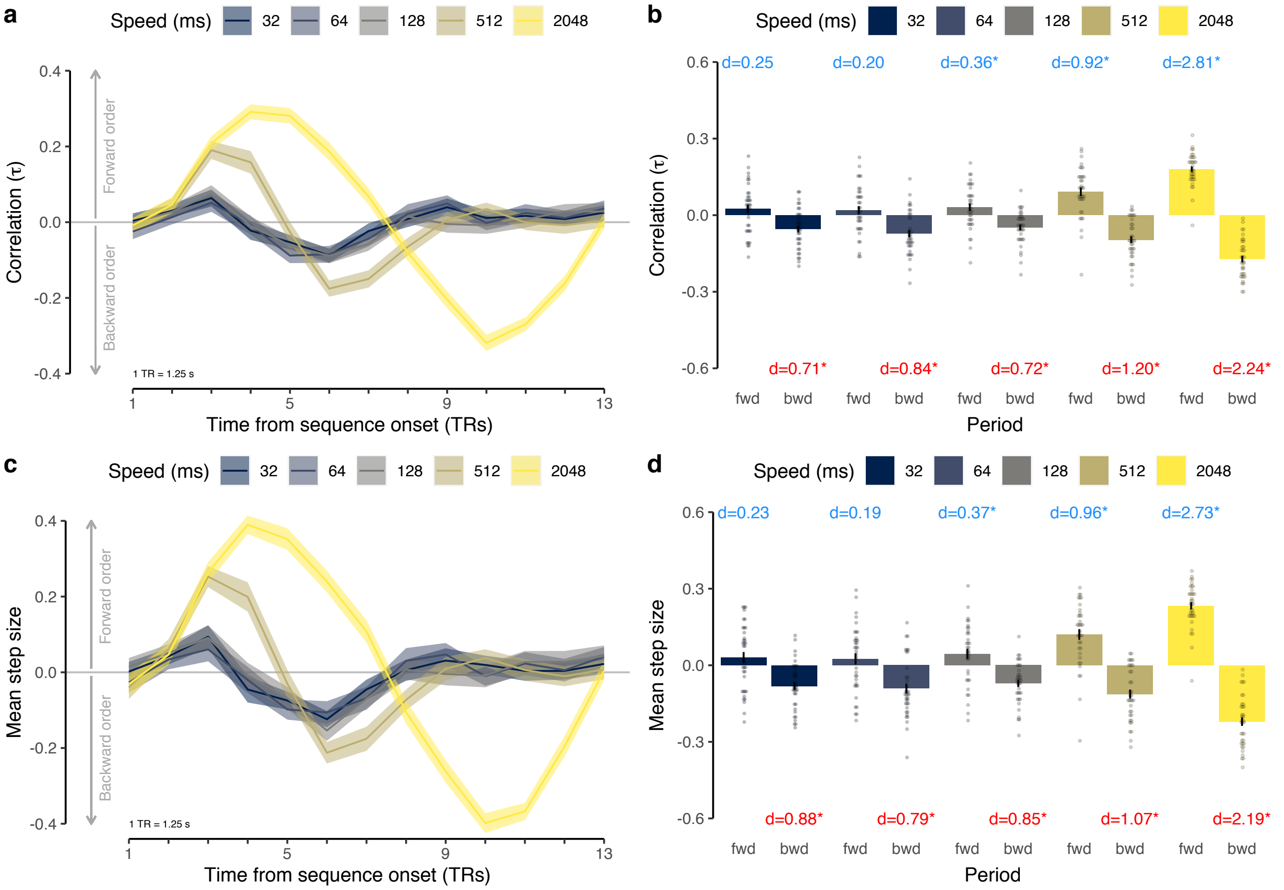

Supplementary Figure S5: **(a)** Time courses (in TRs from sequence onset; x-axis) of mean ranked correlation coefficients between serial event position and classification probabilities (Kendall's τ; y-axis) for each speed condition (in ms; colors) on sequence trials. **(b)** Mean ranked correlation coefficients (Kendall's τ; y-axis) as a function of time period (forward versus backward; x-axis) and sequence speed (in ms; colors; $N = 36$, $ts \geq 2.13$, $ps \leq .05$, $ds \geq 0.36$ (significant tests only), ten two-sided one-sample t-tests against zero, FDR-corrected). **(c)** Time courses (in TRs from sequence onset; x-axis) of the mean step size between probability-ordered within-TR events (y-axis) for each speed condition (in ms; colors) on sequence trials. **(d)** Mean within-TR step-size (y-axis) as a function of time period (forward versus backward; x-axis) and sequence presentation speed (in ms; colors; $N = 36$, $ts \geq 2.25$, $ps \leq .04$, $ds \geq 0.37$ (significant tests only), ten two-sided one-sample t-tests against zero, FDR-corrected). Each dot in (b) and (d) represents averaged data of one participant. Shaded areas in (a), (c) and errorbars in (b), (d) represent mean values ±1 SEM. All statistics have been derived from data of $n = 36$ human participants. 1 TR = 1.25 s. Asterisks indicate significant differences from baseline. Source data are provided as a Source Data file.

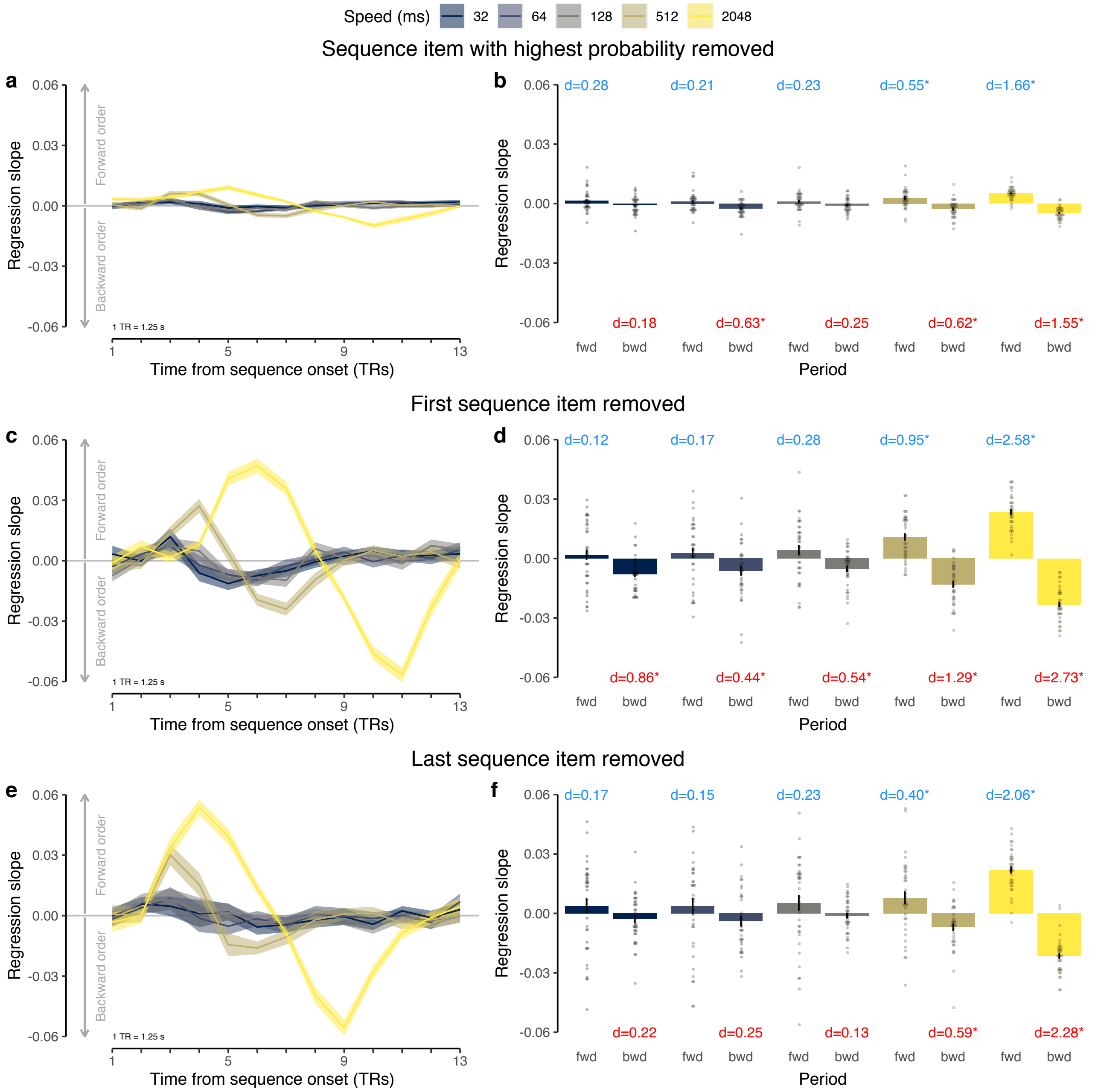

Supplementary Figure S7: Effects of sequence item removal on sequentiality metrics. (a, c, e) Time courses (in TRs from sequence onset; x-axis) of mean slope coefficients of a linear regression between serial event position and classifier probability (y-axis) for each speed condition (in ms; colors) on sequence trials after removal of (a) the sequence item with the highest classification probability, (c) the first sequence item, (e) the last sequence item. **(b, d, f)** Mean slope coefficients (y-axis) as a function of time period (forward versus backward; x-axis) and sequence speed (in ms; colors) after removal of (b) the sequence item with the highest classification probability, (d) the first sequence item, (f) the last sequence item ($N = 36$, ten two-sided one-sample t-tests against zero for each panel, FDR-corrected). Each dot represents averaged data of one participant. Shaded areas in (a, c, e) and errorbars in (b, d, f) represent ± 1 SEM. All statistics have been derived from data of $n = 36$ human participants. 1 TR = 1.25 s. Source data are provided as a Source Data file.

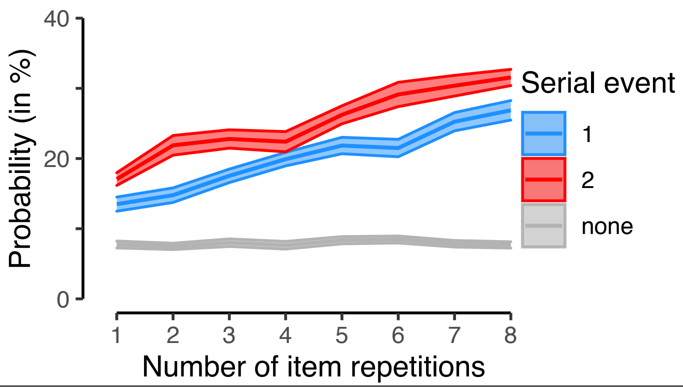

Supplementary Figure S8: Effects of event duration (element repetition) Mean probability (in %; y-axis) as a function of the number of item repetitions (i.e., total event duration), separately for event types (first, second, and out-of-sequence events; colors) based on data of all TRs. Shaded areas represent ±1 SEM. All statistics have been derived from data of $n = 36$ human participants. Source data are provided as a Source Data file.

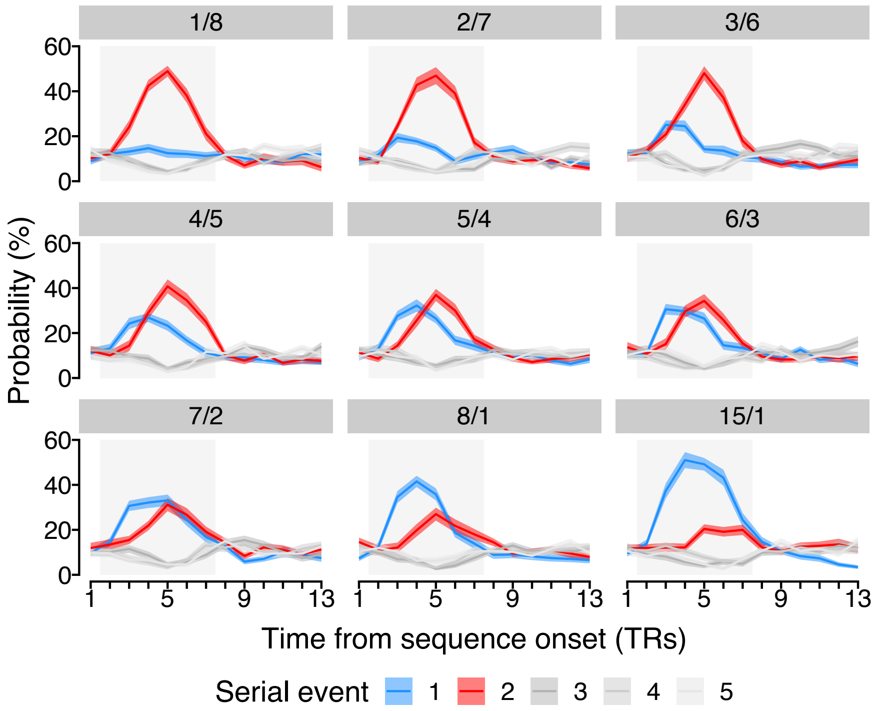

Supplementary Figure S9: Time courses of probabilistic classifier evidence for all repetition conditions. Time courses (in TR from sequence onset; x-axis) of probabilistic classifier evidence (in %; y-axis) on repetition trials grouped by event type (colors), separately for each repetition condition (gray panels). Each panel indicates the number of repetitions per sequence event (e.g., the top-left panel indicates 1 versus 8 repeats of the first versus second event). Time-courses of classifier evidence for the first and second event are shown in blue and red, respectively, while all other stimuli that were not part of the sequence are shown in three shades of gray. Shaded areas represent ±1 SEM. All statistics have been derived from data of $n = 36$ human participants. 1 TR = 1.25 s. Source data are provided as a Source Data file.

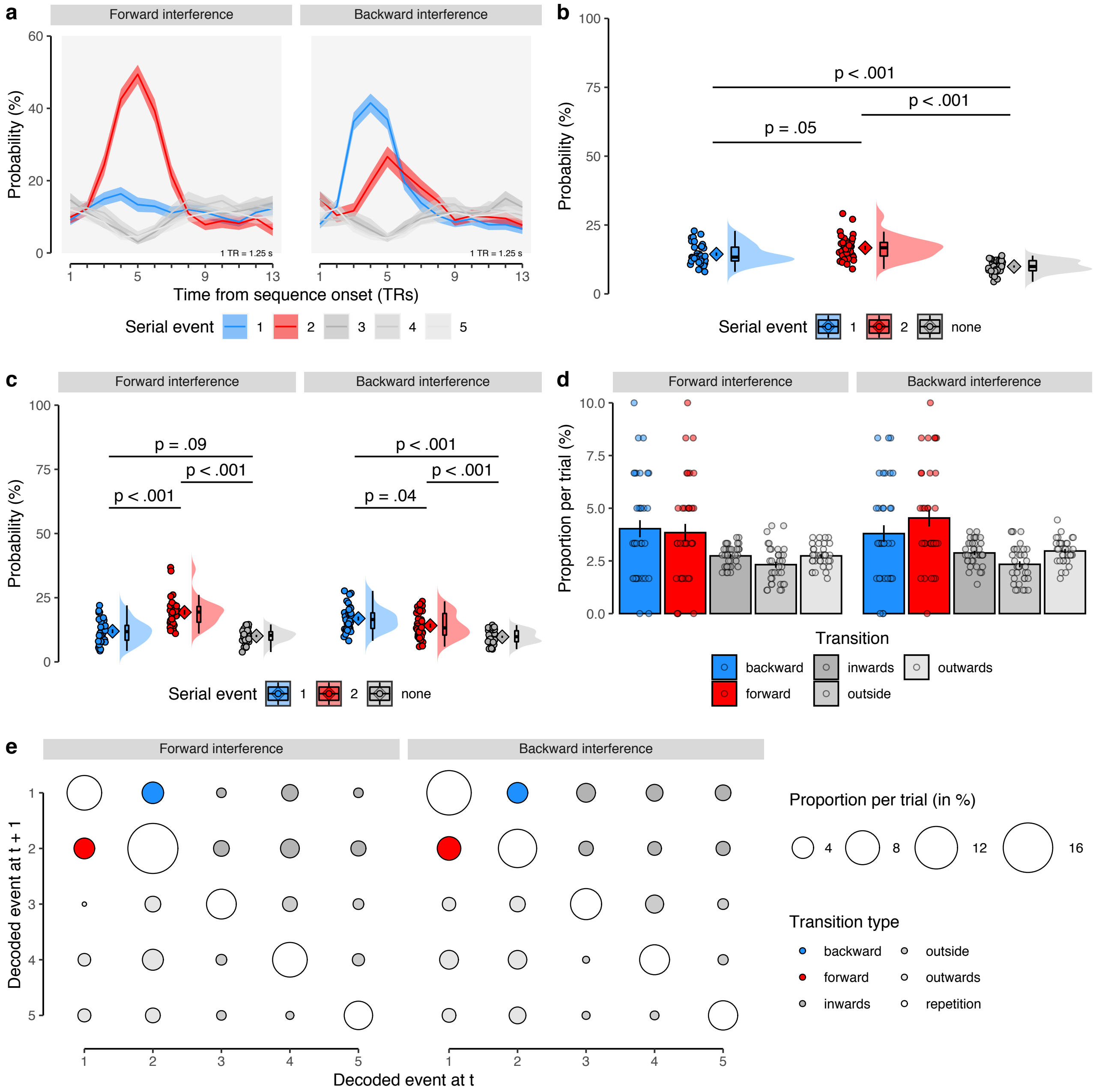

Supplementary Figure S10: Ordering of two-item pairs on repetition trials across all TRs. **(a)** Time courses (in TRs from sequence onset; x-axis) of probabilistic classifier evidence (in %) in repetition trials, color-coded by event type (first/second/non-sequence, see legend). Data shown separately for forward (left) and backward (right) interference conditions. Gray background indicates relevant time period across all TRs. Shaded areas represent ±1 SEM. All statistics have been derived from data of $n = 36$ human participants. 1 TR = 1.25 s. **(b)** Mean probability of event types averaged across all TRs in the relevant time period, as in (a). Each dot represents one participant, the probability density of the data is shown as `rain cloud plots` [cf. 61]. Boxplots indicate the median and interquartile range (IQR). The lower and upper hinges correspond to the first and third quartiles (the 25th and 75th percentiles). The upper whisker extends from the hinge to the largest value no further than 1.5∗ IQR from the hinge (where IQR is the inter-quartile range, or distance between the first and third quartiles). The lower whisker extends from the hinge to the smallest value at most 1.5∗ IQR of the hinge. The barplots show the sample mean and errorbars indicate ±1 SEM. $N = 36$, $ts \geq 2.49$, $ps \leq .045$), LME model with post-hoc Tukey's HSD tests. **(c)** Average probability of event types, separately for conditions as in (a), plots as in (b). ($N = 36$, $ts \geq 2.11$, $ps < .09$), LME model with post-hoc Tukey's HSD tests). **(d)** Mean trial-wise proportion of each transition type, separately for forward/backward conditions, as in (a) ($N = 36$, $ts \geq 2.61$, $ps \leq .05$, four two-sided paired t-tests, Bonferroni-corrected). **(e)** Transition matrix of decoded images indicating mean proportions per trial, separately for the forward and backward condition (left/right). Transition types highlighted in colors (see legend). All statistics have been derived from data of $n = 36$ human participants who participated in one experiment. Source data are provided as a Source Data file.
