# Supplementary material for: Dynamics of fMRI patterns reflect sub-second activation sequences and reveal replay in human visual cortex
Source: Nat Commun. 2021 Mar 19;12:1795. doi: 10.1038/s41467-021-21970-2 (PMC7979874; doi:10.1038/s41467-021-21970-2)
Supplement: Supplementary file 3 — Reporting summary [file 41467_2021_21970_MOESM3_ESM.pdf]

nature research

Corresponding author(s): Nicolas W. Schuck

Last updated by author(s): Dec 9, 2020

Reporting Summary

Nature Research wishes to improve the reproducibility of the work that we publish. This form provides structure for consistency and transparency in reporting. For further information on Nature Research policies, see our Editorial Policies and the Editorial Policy Checklist.

Statistics

For all statistical analyses, confirm that the following items are present in the figure legend, table legend, main text, or Methods section.

n/a	Confirmed	
☐	☒	The exact sample size (*n*) for each experimental group/condition, given as a discrete number and unit of measurement
☐	☒	A statement on whether measurements were taken from distinct samples or whether the same sample was measured repeatedly
☐	☒	The statistical test(s) used AND whether they are one- or two-sided<br>*Only common tests should be described solely by name; describe more complex techniques in the Methods section.*
☐	☒	A description of all covariates tested
☐	☒	A description of any assumptions or corrections, such as tests of normality and adjustment for multiple comparisons
☐	☒	A full description of the statistical parameters including central tendency (e.g. means) or other basic estimates (e.g. regression coefficient) AND variation (e.g. standard deviation) or associated estimates of uncertainty (e.g. confidence intervals)
☐	☒	For null hypothesis testing, the test statistic (e.g. *F*, *t*, *r*) with confidence intervals, effect sizes, degrees of freedom and *P* value noted<br>*Give P values as exact values whenever suitable.*
☒	☐	For Bayesian analysis, information on the choice of priors and Markov chain Monte Carlo settings
☐	☒	For hierarchical and complex designs, identification of the appropriate level for tests and full reporting of outcomes
☐	☒	Estimates of effect sizes (e.g. Cohen's *d*, Pearson's *r*), indicating how they were calculated

Our web collection on statistics for biologists contains articles on many of the points above.

Software and code

Policy information about availability of computer code

Data collection	Behavioral data were collected using a custom task programmed in the commercial software MATLAB [version R2012b; Natick, Massachusetts, USA; The MathWorks Inc.] using the open source Psychophysics Toolbox extensions (version 3.0.11; Brainard, 1997, Spatial Vision; Kleiner et al., 2007, Cognitive and Computational Psychophysics; Pelli, 1997, Spatial Vision) and run on a Windows XP computer. The custom code is publicly accessible at https://github.com/lnnrtwttkhn/highspeed-task and registered on https://zenodo.org/ with DOI: 10.5281/zenodo.4305888. MRI data were acquired using a 32-channel head coil on a 3-Tesla Siemens Magnetom TrioTim MRI scanner (Siemens, Erlangen, Germany).
Data analysis	MRI data were arranged according to the Brain Imaging Data Structure (BIDS; Gorgolewski et al., 2016, Scientific Data) using the HeuDiConv tool (version 0.6.0.dev1; <https://github.com/nipy/heudiconv>). Dicoms were converted to the NIfTI-1 format using dcm2niix (version 1.0.20190410 GCC6.3.0; Li et al., 2016, Journal of Neuroscience Methods). Facial features were eliminated from structural images using pydeface (version 2.0; <https://github.com/poldracklab/pydeface>). The data quality of all functional and structural MRI acquisitions were evaluated using MRIQC (version 0.15.2rc1; Esteban et al., 2018, PLoS One). Preprocessing of BIDS-converted MRI data was performed using fMRIPrep (version 1.2.2; Esteban et al., 2018, Nature Methods; Esteban et al., 2019, Zenodo; RRID:SCR_016216). For more details on the fMRIPrep pipeline, please see <https://fmriprep.readthedocs.io/en/1.2.2/workflows.html> and the Methods section of the manuscript. The fMRI data were spatially smoothed using a Gaussian mask with a standard deviation (FWHM parameter) set to 4 mm using a Nipype (version 1.4.0; Gorgolewski et al., 2011, Frontiers in Neuroinformatics; Gorgolewski et al., 2019, Zenodo) smoothing workflow based on the SUSAN algorithm implemented in the FMRIB Software Library (FSL; Smith & Brady, 1997, International Journal of Computer Vision). All fMRI pattern classification analyses were conducted using open-source packages from the Python (version 3.7; Python Software Foundation, Python Language Reference) modules Nilearn (version 0.5.0; Abraham et al., 2014, Frontiers in Neuroinformatics) and scikit-learn (version 0.20.3; Pedregosa et al., 2011, Journal of Machine Learning Research). Feature selection was performed by combining functional ROIs based on thresholded t-maps with anatomical masks of predefined brain regions. Functional ROIs were based on standard first-level GLMs using SPM12 (version 12.7219; <https://www.fil.ion.ucl.ac.uk/spm/software/spm12/>) running inside a Singularity container built using neurodocker (version 0.7.0; <https://github.com/ReproNim/neurodocker>) implemented in a custom analysis workflow using Nipype (version 1.4.0; Gorgolewski et al., 2011, Frontiers in Neuroinformatics; Gorgolewski et al., 2019, Zenodo). Anatomical masks were created based on

automated anatomical labeling of brain surface reconstructions from T1-weighted reference images created with Freesurfer's recon-all (Dale et al., 1999, NeuroImage) as part of the fMRIPrep workflow. Main statistical analyses were conducted using LME models employing the lmer function of the lme4 package (version 1.1.21, Bates et al., 2015, Journal of Statistical Software) implemented in custom code in R (version 3.6.1, R Core Team, 2019). Model fitting was performed using NLoptr, an R interface to the NLopt library for nonlinear optimization (Johnson et al., 2019) employing the COBYLA (Constrained Optimization BY Linear Approximation) algorithm (Powell, 1994, Springer; Powell, 1998, Acta Numerica). An overview of all custom code used in the study is available on the project website https://wittkuhn.mpib.berlin/highspeed/ and the corresponding GitLab repository at https://git.mpib-berlin.mpg.de/wittkuhn/highspeed/. Please see the data availability statement below for the details on the custom code used to analyze the individual datasets.

For manuscripts utilizing custom algorithms or software that are central to the research but not yet described in published literature, software must be made available to editors and reviewers. We strongly encourage code deposition in a community repository (e.g. GitHub). See the Nature Research guidelines for submitting code & software for further information.

Data

Policy information about availability of data

All manuscripts must include a data availability statement. This statement should provide the following information, where applicable:

- Accession codes, unique identifiers, or web links for publicly available datasets
- A list of figures that have associated raw data
- A description of any restrictions on data availability

We publicly share all code and data used in this study. An overview of all the resources is publicly available on our project website: https://wittkuhn.mpib.berlin/highspeed/. The source code of the website is available at https://git.mpib-berlin.mpg.de/wittkuhn/highspeed. All individual datasets can be found at https://gin.g-node.org/lnnrtwttkhn. Please note, that each dataset is associated with a unique URL and Digital Object Identier (DOI). Data and code management was realized using DataLad [version 0.13.0; 142, for details, see https://www.datalad.org/]. Please note, that instead of separate data and code repositories, we share all data in modularized units alongside the code that created the data, usually in a dedicated code directory in each dataset. This approach allows to better establish the provenance of data (i.e., a better understanding which code produced which data), loosely following the DataLad YODA principles (for details, see the chapter "YODA: Best practices for data analyses in a dataset" in the DataLad handbook [version 0.13; 143], available at https://handbook.datalad.org/).

In particular, we share the following resources: All MRI and behavioral data adhering to the BIDS standard [cf. 102] (https://github.com/lnnrtwttkhn/highspeed-bids; https://gin.g-node.org/lnnrtwttkhn/highspeed-bids; DOI: 10.12751/g-node.4ivuv8), all MRI quality metrics and reports based on MRIQC [cf. 106] (https://github.com/lnnrtwttkhn/highspeed-mriqc; https://gin.g-node.org/lnnrtwttkhn/highspeed-mriqc; DOI: 10.12751/g-node.0vmyuh), all preprocessed MRI data using fMRIPrep [cf. 97, 144] (https://github.com/lnnrtwttkhn/highspeed-fmriprep; https://gin.g-node.org/lnnrtwttkhn/highspeed-fmriprep; DOI: 10.12751/gnode.0ft06t), all binarized anatomical masks used for feature selection (https://github.com/lnnrtwttkhn/highspeed-masks; https://gin.g-node.org/lnnrtwttkhn/highspeed-masks; DOI: 10.12751/g-node.omirok), all first-level GLM results used for feature selection (https://github.com/lnnrtwttkhn/highspeed-glm; https://gin.g-node.org/lnnrtwttkhn/highspeed-glm; DOI: 10.12751/g-node.d21zpv), all results of the multivariate decoding approach (https://github.com/lnnrtwttkhn/highspeed-decoding; https://gin.g-node.org/lnnrtwttkhn/highspeed-decoding; DOI: 10.12751/g-node.9zft1r), all code for the statistical analyses of the results of the decoding analyses (https://github.com/lnnrtwttkhn/highspeed-analysis; https://gin.g-node.org/lnnrtwttkhn/highspeed-analysis; DOI: 10.12751/g-node.eqqdtg), all code to run the behavioral task (https://github.com/lnnrtwttkhn/highspeed-task; DOI: 10.5281/zenodo.4305888), the unprocessed data of the behavioral task acquired during MRI acquisition (https://github.com/lnnrtwttkhn/highspeed-data-behavior; https://gin.g-node.org/lnnrtwttkhn/highspeed-data-behavior; DOI: 10.12751/g-node.p7dabb). Source Data to reproduce the main parts of all figures are provided with this paper.

Field-specific reporting

Please select the one below that is the best fit for your research. If you are not sure, read the appropriate sections before making your selection.

☒ Life sciences ☐ Behavioural & social sciences ☐ Ecological, evolutionary & environmental sciences

For a reference copy of the document with all sections, see nature.com/documents/nr-reporting-summary-flat.pdf

Life sciences study design

All studies must disclose on these points even when the disclosure is negative.

Sample size	No statistical methods were used to predetermine the sample size but it was chosen to be larger than similar previous neuroimaging studies (e.g., Schuck & Niv, 2019, Science; Momennejad et al., 2018, eLife; Tambini et al., 2013, PNAS).
Data exclusions	Four participants were excluded from further analysis because their mean behavioral performance was below the 50% chance level in either or both the sequence and repetition trials suggesting that they did not adequately process the visual stimuli used in the task. This exclusion criterion was pre-established based on previous piloting results of the behavioral task.
Replication	No direct replication of the experiment was performed. The current study is itself a conceptual replication of Schuck & Niv, 2019, Science.
Randomization	For the task condition that showed sequences of five images we ensured that all possible sequences were chosen equally often across all participants. Given 120 possible sequential combinations in total and 15 sequences per participant, the sequences were distributed across eight groups of participants. Sequences were randomly assigned to each participant following this pseudo-randomized procedure.
Blinding	Blinding is not relevant to our study as we treat data from all participants equally.

Reporting for specific materials, systems and methods

We require information from authors about some types of materials, experimental systems and methods used in many studies. Here, indicate whether each material, system or method listed is relevant to your study. If you are not sure if a list item applies to your research, read the appropriate section before selecting a response.

Materials & experimental systems

n/a	Involved in the study
☒	☐ Antibodies
☒	☐ Eukaryotic cell lines
☒	☐ Palaeontology and archaeology
☒	☐ Animals and other organisms
☐	☒ Human research participants
☒	☐ Clinical data
☒	☐ Dual use research of concern

Methods

n/a	Involved in the study
☒	☐ ChIP-seq
☒	☐ Flow cytometry
☐	☒ MRI-based neuroimaging

Human research participants

Policy information about studies involving human research participants

Population characteristics	The final sample consisted of 36 participants (mean age = 24.61 years, SD = 3.77 years, age range: 20 - 35 years, 20 female, 16 male). All participants were screened for MRI eligibility during a telephone screening prior to participation and again at the beginning of each study session according to standard MRI safety guidelines (e.g., asking for metal implants, claustrophobia, etc.). None of the participants reported to have any major physical or mental health problems. All participants were required to be right-handed, to have corrected-to-normal vision, and to speak German fluently. Furthermore, only participants with a head circumference of 58 cm or less could be included in the study. This requirement was necessary as participants' heads had to fit the MRI head coil together with MRI-compatible headphones that were used during the experimental tasks.
Recruitment	Participants were recruited from an internal participant database or through local advertisement. Any potential self-selection bias, if present, cannot be explicitly ruled out since participants freely chose whether they wanted to participate and contact the experimenter based on the public advertisement and announcements sent through the participant database. These biases are, if present, unlikely to affect the results since the experiment was conducted in a within-subjects design (i.e., all participants experienced all conditions).<br>The pseudo-randomized procedure used to assign all 120 possible stimulus sequences to participants (see above section on "Randomization") is also unlikely to interact with any self-selection bias, if present.<br>The main effects investigated in this study (fMRI patterns of fast activation sequences) can be considered general and not specific to a population of young and healthy individuals with high education.
Ethics oversight	The ethics commission of the German Psychological Society (DGPs) approved the study protocol (Reference number: NS 012018).

Note that full information on the approval of the study protocol must also be provided in the manuscript.

Magnetic resonance imaging

Experimental design

Design type	The study consisted of two experimental sessions. In each session, we acquired four functional task runs of about 11 minutes during which participants performed the main task in an event-related design. We also recorded two functional runs of resting-state fMRI data, one before and one after the task runs.
Design specifications	In each session, we acquired four functional task runs of about 11 minutes during which participants performed the main task in an event-related task design. During each functional run, participants performed trials from three different task conditions. Slow trials started with a waiting period of 3.85 s during which a blank screen was presented. After a fixation dot for 300 ms, a stimulus was shown for 500 ms followed by a variable ISI during which a blank screen was presented again. The duration of the ISI was drawn from a truncated exponential distribution with a mean of 2.5 s and a lower limit of 1 s. Behavioral responses were collected during a fixed time period of 1.5 s after each stimulus onset. During sequence and repetition trials a target cue was shown for 1000 ms followed by a blank screen for 3850 ms. A short presentation of a gray fixation dot for 300 ms signaled the onset of the upcoming sequence of visual objects. All objects in the sequence were presented briefly for 100 ms. The ISI for each trial was determined based on the current sequence speed (32, 64, 128, 512, or 2048 ms) and was the same for all stimuli within a sequence. The sequence of stimuli was followed by a delay period with a gray fixation dot that was terminated once 16 s since the onset of the first sequence object had elapsed. Subsequently, the name of the target object as well as the response mapping were presented for 1.5 s. Slow trials were interleaved with sequence and repetition trials such that each of the 120 slow trials was followed by either one of the 75 sequence trials or 45 repetition trials. For resting state scans, participants were asked to stay awake and focus on a white fixation cross presented centrally on a black screen. Each resting-state run was about 5 minutes in length, during which 233 functional volumes were acquired.
Behavioral performance measures	Behavioral performance was assessed by measuring correct button presses (accuracy) and response times. Mean behavioral accuracy was used as the primary indicator to establish that the subjects were performing the task as expected.

Acquisition

Imaging type(s)

Functional and structural imaging

Field strength

3-Tesla

Sequence & imaging parameters

For the functional scans, whole-brain images were acquired using a segmented k-space and steady state T2*--weighted multiband (MB) echo-planar imaging (EPI) single-echo gradient sequence that is sensitive to the blood-oxygen-level dependent (BOLD) contrast (sequence specification: 64 slices in interleaved ascending order; anterior-to-posterior (A-P) phase encoding direction; repetition time (TR) = 1250 ms; echo time (TE) = 26 ms; voxel size = 2 x 2 x 2 mm; matrix = 96 x 96; field of view (FOV) = 192 x 192 mm; flip angle (FA) = 71 degrees; distance factor = 0%; MB acceleration factor 4). Slices were tilted for each participant by 15 degrees forwards relative to the rostro-caudal axis to improve the quality of fMRI signal from the hippocampus (cf. Weiskopf et al., 2006, NeuroImage) while preserving good coverage of occipito-temporal brain regions. These sequence parameters were the same for task and resting state acquisitions. After the functional task and resting-state runs, two short acquisitions with six volumes each were collected using the same sequence parameters as for the functional scans but with varying phase encoding polarities, resulting in pairs of images with distortions going in opposite directions between the two acquisitions which were used for distortion correction. In addition, a whole-brain spoiled gradient recalled (GR) field map with dual echo-time images (sequence specification: 36 slices; A-P phase encoding direction; TR = 400 ms; TE1 = 4.92 ms; TE2 = 7.38 ms; FA = 60 degrees; matrix size = 64 x 64; FOV = 192 x 192 mm; voxel size = 3 x 3 x 3.75 mm) was obtained. High-resolution T1-weighted anatomical Magnetization Prepared Rapid Gradient Echo (MPRAGE) sequences were obtained from each participant to allow registration and brain surface reconstruction (sequence specification: 256 slices; TR = 1900 ms; TE = 2.52 ms; FA = 9 degrees; inversion time (TI) = 900 ms; matrix size = 192 x 256; FOV = 192 x 256 mm; voxel size = 1 x 1 x 1 mm).

Area of acquisition

Whole-brain images were acquired.

Diffusion MRI ☐ Used ☒ Not used

Preprocessing

Preprocessing software

Preprocessing of BIDS-converted MRI data was performed using fMRIPrep (version 1.2.2; Esteban et al., 2018, Nature Methods; Esteban et al., 2019, Zenodo; RRID:SCR_016216). fMRIPrep uses a combination of tools from neuroimaging software packages, including FSL, ANTs, Freesurfer and AFNI. Details of the pipeline are reported in the main manuscript based on fMRIPrep's citation boilerplate that is published together with the the preprocessed MRI data (please see section on Data Availability) and can also be found at https://fmriprep.readthedocs.io/en/1.2.2/workflows.html. The fMRI data were spatially smoothed using a Gaussian mask with a standard deviation (FWHM parameter) set to 4 mm using a Nipype (version 1.4.0; Gorgolewski et al., 2011, Frontiers in Neuroinformatics; Gorgolewski et al., 2019, Zenodo) smoothing workflow based on the SUSAN algorithm implemented in the FMRIB Software Library (FSL; Smith & Brady, 1997, International Journal of Computer Vision).

Normalization

Spatial normalization to the ICBM 152 Nonlinear Asymmetrical template version 2009c (RRID:SCR_008796; Fonov et al., 2009, NeuroImage) was performed through nonlinear registration with antsRegistration (Advanced Normalization Tools (ANTs); version 2.2.0; RRID:SCR_004757; Avants et al., 2008, Medical Image Analysis), using brain-extracted versions of both T1-weighted volume and template. All classification analyses were performed on functional images that were co-registered to the individual T1-weighted reference. Here, a reference volume and its skull-stripped version were generated using a custom methodology of fMRIPrep. The BOLD reference was then co-registered to the T1-weighted reference using bbregister (FreeSurfer) which implements boundary-based registration (Greve et al., 2009, NeuroImage). Co-registration was configured with nine degrees of freedom to account for distortions remaining in the BOLD reference.

Normalization template

The ICBM 152 Nonlinear Asymmetrical template version 2009c (RRID:SCR_008796; Fonov et al., 2009, NeuroImage) was used for nonlinear normalization.

Noise and artifact removal

We included the following nuisance regressors estimated during preprocessing with fMRIPrep in the first-level GLMs used to determine the functional ROIs for feature selection: the frame-wise displacement for each volume as a quantification of the estimated bulk-head motion, the six rigid-body motion-correction parameters estimated during realignment (three translation and rotation parameters, respectively), and six noise components calculated according to the anatomical variant of CompCorr (Esteban et al., 2018, Nature Methods).

Volume censoring

No volume censoring was performed.

Statistical modeling & inference

Model type and settings

The main analyses were performed using multivariate leave-one-run-out cross-validated pattern classification. Feature selection was performed by combining functional ROIs based on thresholded t-maps with anatomical masks of predefined brain regions. Details about the multivariate classification analysis and the creation of functional ROIs can be found in the section "Multivariate modeling and predictive analysis" below. Details about the creation of anatomical ROIs can be found in the section "Anatomical location(s)" below.

Effect(s) tested

The following effects were tested: Behavioral accuracy in the three task conditions compared to chance; cross-validated classification accuracy on slow trials compared to chance in occipito-temporal and hippocampal data; peak in classification probabilities of the true class on slow trials compared to all other classes and chance in occipito-temporal and hippocampal data; mean TR-wise linear regression slope coefficients as a function of sequence speed and time period compared to chance, Pearson's correlation between time courses of regression slopes predicted by modeling approach and based on data between and within participants; mean serial event position as a function of sequence speed and time period compared to

chance; mean step size between decoded events as a function of sequence speed, time period and early versus late phases of time periods compared to chance; mean classification probabilities of repetition events as a function of event order and number of repetition using LME models; mean proportion of transition types on repetition trials as a function of interference condition; standard deviation and absolute mean of regression slopes in fast versus slow sequence and resting state data using t-tests; frequency spectrum analysis of TR-wise regression slope coefficients in fast and slow sequence data compared to resting state and sequence-inserted resting state data; changes in standard deviation and frequency power as a function of number, SNR level and speed of inserted sequence data compared to sequence-free resting state data; frequency spectrum analysis of TR-wise regression slope coefficients in pre- and post-task resting state data assuming all 120 possible order permutations of sequential stimuli.

Specify type of analysis: ☐ Whole brain ☒ ROI-based ☐ Both

Anatomical location(s): All participant-specific anatomical masks were created based on automated anatomical labeling of brain surface reconstructions from the individual T1-weighted reference image created with Freesurfer's recon-all (Dale, 1999, NeuroImage) as part of the fMRIPrep workflow (Esteban, 2018, Nature Methods). For the anatomical masks of occipito-temporal regions we selected the corresponding labels of the cuneus, lateral occipital sulcus, pericalcarine gyrus, superior parietal lobule, lingual gyrus, inferior parietal lobule, fusiform gyrus, inferior temporal gyrus, parahippocampal gyrus, and the middle temporal gyrus (cf. Haxby et al., 2001, Science). For anatomical masks of the hippocampus we selected the label corresponding to the hippocampus.

Statistic type for inference (See Eklund et al. 2016): Average or ROI-based tests

Correction: Corrections for multiple comparisons were performed by controlling the false discovery rate (FDR) and using the Bonferroni correction.

Models & analysis

n/a	Involved in the study
☒	☐ Functional and/or effective connectivity
☒	☐ Graph analysis
☐	☒ Multivariate modeling or predictive analysis

Multivariate modeling and predictive analysis

We used separate multinomial logistic regression classifiers with identical parameter settings. All classifiers were regularized using L2 regularization. The C parameter of the cost function was fixed at the default value of 1.0 for all participants. The classifiers employed the lbfgs algorithm to solve the multi-class optimization problem and were allowed to take a maximum of 4,000 iterations to converge. Pattern classification was performed within each participant separately, never across participants. For each stimulus in the training set, we added 4 s to the stimulus onset and chose the volume closest to that time point (i.e., rounded to the nearest volume) to center the classifier training on the expected peaks of the BOLD response (for a similar approach, see e.g. Deuker, 2013, Journal of Neuroscience). At a TR of 1.25 s this corresponded to the fourth MRI volume which thus compromised a time window of 3.75 s to 5 s after each stimulus onset. We detrended the fMRI data separately for each run across all task conditions to remove low frequency signal intensity drifts in the data due to noise from the MRI scanner. For each classifier and run, the features were standardized (z-scored) by removing the mean and scaling to unit variance separately for each test set.

Feature selection was performed by combining a functional ROI approach based on thresholded t-maps with anatomical masks to select image-responsive voxels within a predefined anatomical brain region. Functional ROIs were based on standard first-level GLMs using SPM12 (version 12.7219; <https://www.fil.ion.ucl.ac.uk/spm/software/spm12/>) running inside a Singularity container built using neurodocker (version 0.7.0; <https://github.com/ReproNim/neurodocker>) implemented in a custom analysis workflow using Nipype (version 1.4.0; Gorgolewski et al., 2011, Frontiers in Neuroinformatics; Gorgolewski et al., 2019, Zenodo). In each cross-validation fold, we fitted a first-level GLM to the data in the training set (e.g., data from run 1 to 7) and modeled the stimulus onset of all trials of the slow task when a stimulus was presented upright and was correctly rejected (i.e., participants correctly did not respond). These trial events were modeled as boxcar functions with the length of the modeling event corresponding to the duration of the stimulus on the screen (500 ms for all events). If present in the training data, we also included trials with hits (correct response to upside-down stimuli), misses (missed response to upside-down stimuli) and false alarms (incorrect response to upright stimuli) as regressors of no interest, thereby explicitly modeling variance attributed to these trial types (Mumford et al., 2012, NeuroImage). Finally, we included the following nuisance regressors estimated during preprocessing with fMRIPrep: the frame-wise displacement for each volume as a quantification of the estimated bulk-head motion, the six rigid-body motion-correction parameters estimated during realignment (three translation and rotation parameters, respectively), and six noise components calculated according to the anatomical variant of CompCorr. All regressors were convolved with a canonical HRF and did not include model derivatives for time and dispersion. Serial correlations in the fMRI time series were accounted for using an autoregressive AR(1) model. The resulting brain maps of voxel-specific t-values resulting from the estimation of the described t-contrast were then combined with an anatomical mask of occipito-temporal brain regions (see details above).
